# An efficient process for wastewater treatment to mitigate free nitrous acid generation and its inhibition on biological phosphorus removal

**DOI:** 10.1038/srep08602

**Published:** 2015-02-27

**Authors:** Jianwei Zhao, Dongbo Wang, Xiaoming Li, Qi Yang, Hongbo Chen, Yu Zhong, Hongxue An, Guangming Zeng

**Affiliations:** 1 College of Environmental Science and Engineering, Hunan University, Changsha 410082, China; 2 Key Laboratory of Environmental Biology and Pollution Control, Hunan University, Ministry of Education, Changsha 410082, China; 3 State Key Laboratory of Pollution Control and Resources Reuse, School of Environmental Science and Engineering, Tongji University, 1239 Siping Road, Shanghai 200092, China; 4 Advanced Water Management Centre, The University of Queensland, QLD 4072, Australia; 5 Jiangsu Tongyan Environmental Production Science and Technology Co. Ltd., Yancheng. 224000, China

## Abstract

Free nitrous acid (FNA), which is the protonated form of nitrite and inevitably produced during biological nitrogen removal, has been demonstrated to strongly inhibit the activity of polyphosphate accumulating organisms (PAOs). Herein we reported an efficient process for wastewater treatment, i.e., the oxic/anoxic/oxic/extended-idle process to mitigate the generation of FNA and its inhibition on PAOs. The results showed that this new process enriched more PAOs which thereby achieved higher phosphorus removal efficiency than the conventional four-step (i.e., anaerobic/oxic/anoxic/oxic) biological nutrient removal process (41 ± 7% versus 30 ± 5% in abundance of PAOs and 97 ± 0.73% versus 82 ± 1.2% in efficiency of phosphorus removal). It was found that this new process increased pH value but decreased nitrite accumulation, resulting in the decreased FNA generation. Further experiments showed that the new process could alleviate the inhibition of FNA on the metabolisms of PAOs even under the same FNA concentration.

Enhanced biological phosphorus removal is one important strategy to protect natural waters from eutrophication. It is usually achieved through culturing an activated sludge with alternating anaerobic and oxic conditions, by which polyphosphate accumulating organisms (PAOs), the microorganisms responsible for phosphorus removal in wastewater treatment plants (WWTPs), can be largely enriched. To gain deep understandings regarding this biological phosphorus removal regime, numerous studies have been made in the past two decades^1^,^2^. It is widely accepted that PAOs take up available carbon sources anaerobically and store them as poly-β-hydroxyalkanoates (PHAs), with the energy and reducing power mainly gained through polyphosphate cleavage and glycogen degradation, respectively. In the subsequent oxic phase, the stored PHAs are utilized for cell growth, glycogen replenishment, phosphorus uptake, and polyphosphate accumulation^2^,^3^,^4^. This metabolism behavior is considered to provide a selective advantage to PAOs over other populations.

In general, biological phosphorus removal can be excellently achieved in well-defined laboratory experiments, with the high abundance of PAOs above 90%^5^. In real WWTPs, however, unpredictable failures due to lost or reduced activity of PAOs are often observed^2^. This is primarily because biological phosphorus removal in full-scale WWTPs usually occurs along with biological nitrogen removal, by which denitrifiers will compete with PAOs for the limited carbon sources available in wastewaters, the recycled mixtures will disturb the anaerobic circumstance, and some intermediates of nitrogen removal such as nitrite and free nitrous acid (FNA) will inhibit the metabolisms of PAOs. Among them, the effect of FNA on the metabolisms of PAOs has been drawn much attention recently owing to its strong inhibition on the activities of PAOs^5^,^6^,^7^,^8^.

Nitrite is inevitably produced in substantial amounts during biological nitrogen removal. It was reported that nitrite concentration could accumulate up to 12.3–22.6 mg/L in domestic wastewater treatment processes^9^. Especially in some WWTPs that achieve nitrogen removal via the nitrite pathway, the accumulated concentration could reach up to 40 mg/L^10^. Previous researchers considered that the intermediate of nitrification and denitrification (i.e., nitrite) caused seriously inhibition on the metabolisms of PAOs, but recently there have been increasing evidences showing that FNA, the protonated form of nitrite, rather than nitrite is the actual inhibitor^5^,^11^,^12^. For example, it was reported that FNA could inhibit aerobic phosphorus uptake seriously at a low level of 0.5 × 10^−3^ mg HNO_2_-N/L^11^, and more than 1.5 × 10^−3^ mg HNO_2_-N/L could result in the complete loss of aerobic phosphorus uptake^13^. Pijuan et al.^5^ showed that the aerobic phosphorus uptake was inhibited by 50% when FNA reached 0.52 × 10^−3^ mg HNO_2_-N/L. Anoxic phosphorus uptake was also significantly affected by FNA presence^14^, and 0.02 mg HNO_2_-N/L would cause the complete loss of anoxic phosphorus uptake^11^. Furthermore, anaerobic metabolisms of PAOs were also severely affected by FNA, and Ye et al.^8^ demonstrated that FNA had an adverse effect on carbon source uptake even at 1.0 × 10^−3^ mg HNO_2_-N/L. Due to the severe inhibition on PAOs caused by FNA and the massive quantity of wastewaters treated daily, any improvement for reducing FNA generation or mitigating its inhibition on PAOs in current methods should have tangible economic and ecological consequences.

Several strategies, such as activated sludge adaption, pH adjustment, temperature control, and the feed flow and mode optimization, have been recommended to minimize the inhibitory effect of FNA on PAOs^6^,^15^. Though previous researches have proposed these meaningful methods, the strategy for mitigating the generation of FNA and its inhibition on PAOs from the aspect of modifying wastewater treatment operation regime has never been reported before. In addition, some previously proposed strategies such as pH adjustment and temperature control are rarely or not practically applied in full-scale WWTPs, probably due to the associated costs of adding pH controlling agents or increasing constructions. Thus, the method obtained in terms of wastewater treatment process modification may provide an alternatively practical option for engineers.

Besides the widely accepted anaerobic/oxic (A/O) phosphorus removal regime, PAOs are verified to be also enriched readily in the oxic/extended-idle (O/EI) wastewater treatment regime^16^,^17^,^18^,^19^. The O/EI regime enriches PAOs via some specific metabolic reactions (e.g., a significant idle release of phosphate and a low idle production of PHAs) occurred in the extended-idle phase, which shows a different inducing mechanism from the classical A/O regime. It is also reported that when receiving the same level of nitrate, the transformations of metabolic intermediates (especially the accumulation of nitrite) in the O/EI regime are much lower affected than those in the A/O regime^16^. Thus, one method that might be used to decrease FNA generation or mitigate its inhibition on PAOs from the viewpoint of wastewater treatment regime, we think, is to develop a suitable biological nutrient removal (BNR) process based on the O/EI regime. Although several studies have been performed in terms of the O/EI phosphorus removal regime, the questions as to whether (and how) this regime can achieve good performances of simultaneous nitrogen and phosphorus removal remain unknown. Additionally, it is also unclear whether PAOs cultured in this O/EI based on BNR process can tolerate higher level of disturbances caused by nitrogen removal (e.g., FNA inhibition) than those cultured in the conventional BNR process.

The purpose of this paper is to report this efficient method for significantly mitigating the generation of FNA and its inhibition on PAOs. Firstly, a new BNR process is designed based on the recently exploited O/EI regime, and its feasibility of BNR is evaluated. Since the O/EI regime is a phosphorus removal process with low nitrogen removal performances (around 60%), an anoxic phase is inserted into the oxic phase to enhance nitrogen removal. Therefore, the new BNR process developed here is performed as the oxic/anoxic/oxic/extended-idle (O/A/O/EI) regime. Then, the performances of BNR and the abundances of PAOs between the new process and conventional four-step (i.e., anaerobic/oxic/anoxic/oxic, defined as A/O/A/O) BNR process are compared. Finally, the reasons for the new process showing higher abundance of PAOs are explored via the analysis of cyclic pH variation, nitrite accumulation, and changes of metabolic intermediates in PAO metabolisms.

## Results

### BNR performances in the O/A/O/EI reactor during the long-term operation

**Figure 1:**
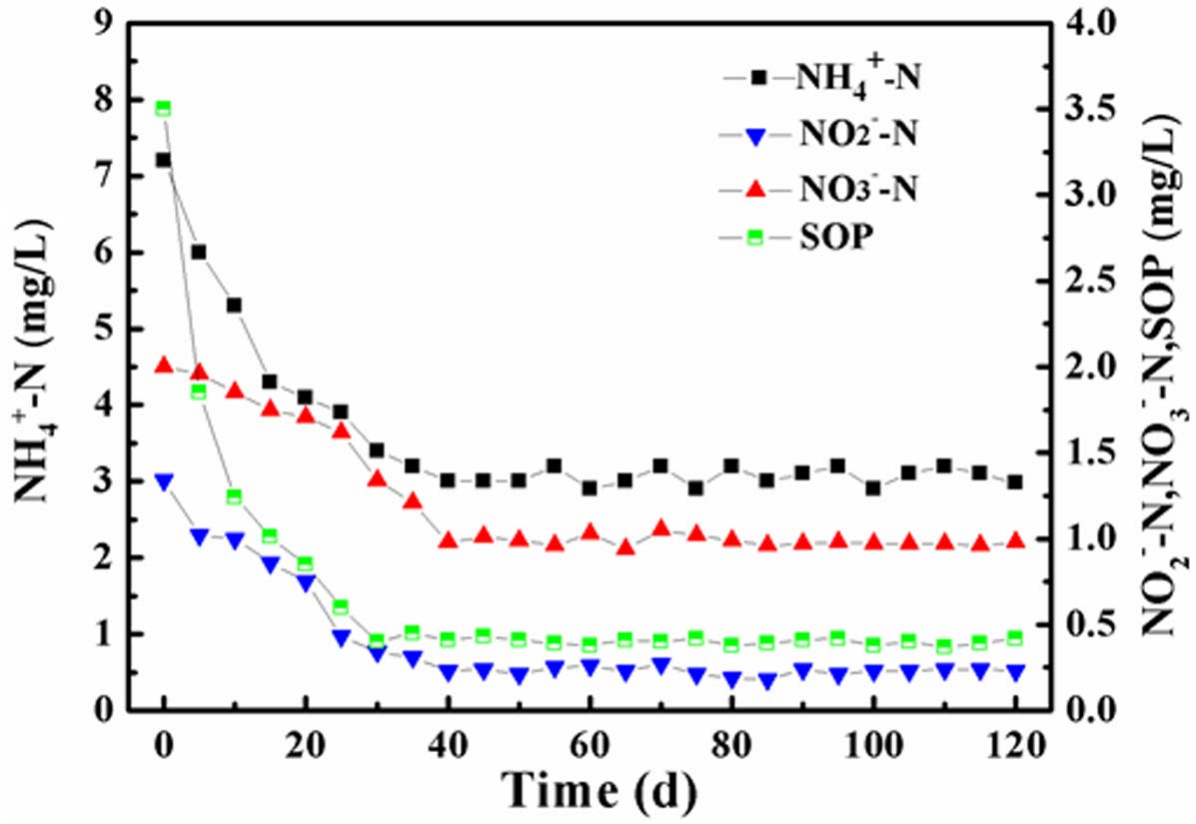
Variations of effluent NH_4_^+^-N, NO_2_^-^-N, NO_3_^-^-N, and SOP in O/A/O/EI reactor during the long-term operation.

The data of the effluent NH_4_^+^-N, NO_2_^-^-N, NO_3_^-^-N, and soluble orthophosphate (SOP) concentrations in the O/A/O/EI reactor during the long-term operation are illustrated in Figure 1. It can be seen that the concentrations of these nutrients in effluent decreased along with the acclimated time. After domestication for about 40 d, the effluent nutrient concentrations became stable. The effluent NH_4_^+^-N, NO_2_^-^-N, NO_3_^-^-N, and SOP concentrations during stable operation were respectively maintained among 2.67–3.49, 0.15–0.31, 0.83–1.25 and 0.30–0.52 mg/L, which indicated that the efficiencies of nitrogen and phosphorus removal in the O/A/O/EI reactor were above 91% and 96%, respectively. The long-term experimental data showed that BNR could be successfully achieved in the new O/A/O/EI process.

### Comparison of BNR performances between the O/A/O/EI and A/O/A/O reactors

**Figure 2:**
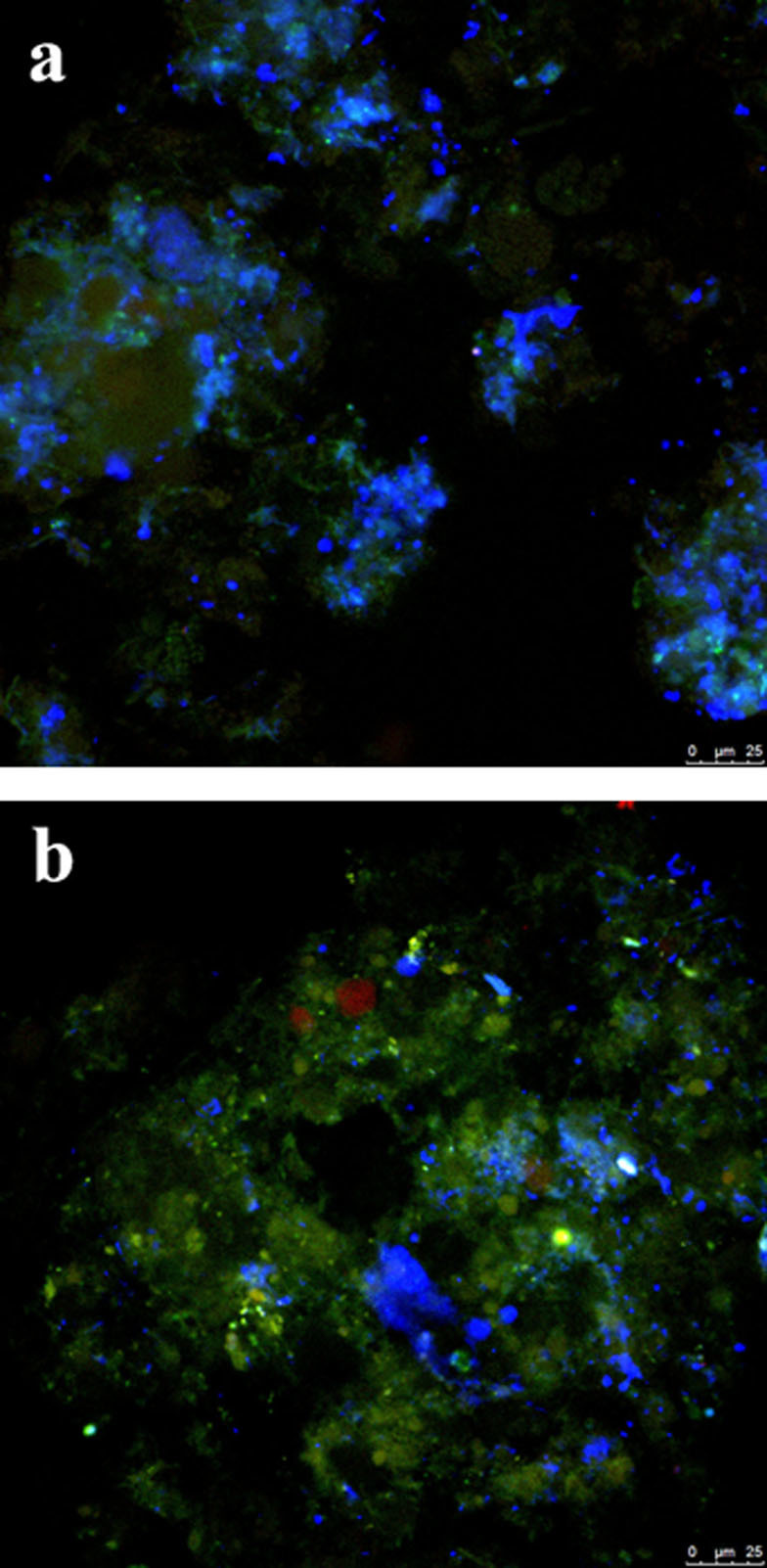
FISH micrographs of microbial communities from O/A/O/EI reactor (a) and A/O/A/O reactor (b) hybridizing with PAOmix (blue), GAOmix (red) and EUBmix(green) probes, respectively. Cells that were yellow had hybridized with both GAOmix and EUBmix probes. Samples were obtained after stable operation (on day 80).

The BNR performances between the O/A/O/EI and A/O/A/O reactors during a 21-day stable operation are summarized in Table 1. It was found that nitrogen removal was not obviously affected by the different operation processes. Although the effluent NH_4_^+^-N, NO_2_^-^-N, and NO_3_^-^-N in the O/A/O/EI reactor were slightly lower than those in the A/O/A/O reactor, the efficiency of nitrogen removal between the two reactors was very close. However, the effluent SOP concentration in the O/A/O/EI reactor were much lower than that in the A/O/A/O reactor (0.41 ± 0.11 mg/L versus 2.70 ± 0.18 mg/L), which thereby caused a much higher phosphorus removal efficiency (97 ± 0.73% versus 82 ± 1.2%). FISH quantification further showed that the abundances of PAOs and glycogen accumulating organisms (GAOs) were respectively accounted for 41 ± 7% and 11 ± 3% in the O/A/O/EI reactor while the corresponding data in the A/O/A/O reactor were 30 ± 5% and 24 ± 4%, respectively (Figure 2), which were consistent with the phosphorus removal efficiency shown in Table 1. The above results clearly displayed that by modifying wastewater treatment operation regime the abundance of PAOs and the efficiency of phosphorus removal could be improved.

### Phosphorus removal test via chemical precipitation at different pH

Batch tests in the absence of activated sludge microorganisms were performed to figure out the effect of pH on chemical phosphorus removal (Table S1, supporting information). As shown in Table S1, negligible SOP removal via chemical precipitation was observed. Only 2% of SOP was removed via chemical precipitation even the pH was 8.5, which implied that phosphorus removal in this study was dominated by biological effect.

### Comparison of the effect of different FNA levels on PAO metabolisms between the two reactors

The two BNR reactors were developed from different inducing mechanisms of biological phosphorus removal. The different inducing mechanisms might give rise to different metabolic responses generated by PAOs even under the same level of FNA, thus we examined whether the same level of FNA would bring different effects on the metabolisms of PAOs between the two reactors. It can be seen from Table 2 that the metabolisms of PAOs in the conventional A/O/A/O reactor were severely inhibited by the FNA addition. When FNA concentration was 0, the effluent SOP in the A/O/A/O reactor was 0.75 ± 0.05 mg/L. With the increased FNA concentration anaerobic SOP release, PHA-up/VFA ratio, Gly-de/VFA ratio, and Gly-syn were significantly decreased. As a result, effluent SOP concentration was increased largely. Especially when FNA was 0.51 × 10^−3^ mg/L, 7.05 ± 0.09 mg/L of SOP was measured in the effluent, suggesting that only 53 ± 0.6% of influent SOP was removed. Also from Table 2, it can be found that the influence of FNA on the metabolisms of PAOs cultured in the O/A/O/EI reactor was weaker than that in the conventional A/O/A/O reactor. Even at FNA concentration of 0.51 × 10^−3^ mg/L, 5.10 ± 0.08 mg/L was determined in the effluent, which indicated about 66 ± 0.5% of influent SOP was removed. Further analysis revealed that compared with 0 mg/L of FNA, 0.51 × 10^−3^ mg/L of FNA caused by 33% decrease in phosphorus removal in the O/A/O/EI reactor, whereas the corresponding datum was 44.2% in the conventional A/O/A/O reactor. Similar observations were also observed in other FNA levels.

## Discussion

### The possible mechanisms of O/A/O/EI regime culturing higher PAO abundance

**Figure 3:**
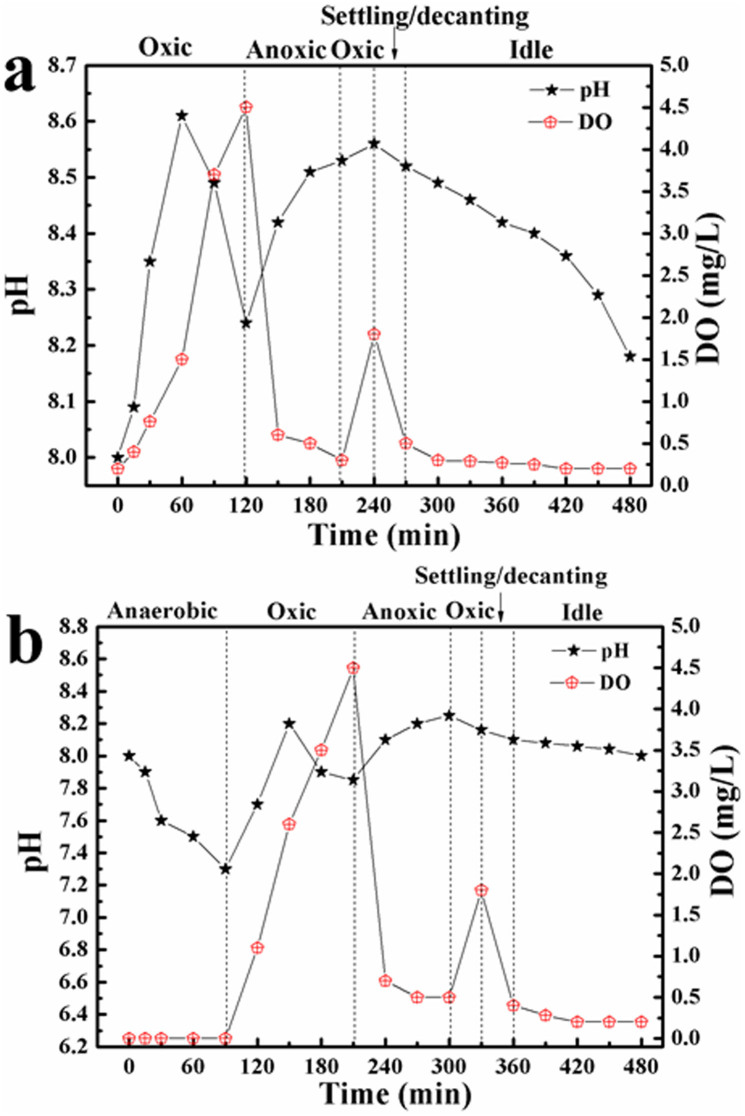
Variations of pH and DO in one typical cycle on Day 80 (a: O/A/O/EI reactor; b: A/O/A/O reactor).

Several parameters can affect significantly the abundance of PAOs. By comparing the operational conditions between the two reactors, dissolved oxygen (DO) and pH might be the effect parameters since they are not constantly controlled during the whole process. Therefore, the cyclic variations of DO and pH between the two reactors during the steady-state operation were first compared, and the data are shown in Figure 3. Except for the anaerobic phase DO concentration in other phases of the A/O/A/O reactor showed very similar changes with that in the O/A/O/EI reactor. For example, in the experiment of day 80, DO in the O/A/O/EI reactor kept low levels during the initial period of first oxic phase and then gradually increased to a final concentration of 4.5 mg/L at the end of first oxic phase. During the subsequent anoxic phase, DO decreased rapidly to 0.7 mg/L and kept in the range of 0.4–0.7 mg/L in the remainder of anoxic phase. In the second oxic phase, DO increased gradually to 1.8 mg/L. After that, DO decreased gradually to 0.3 mg/L during the initial 60 min of idle phase and further decreased to 0.2 mg/L during the remainder of idle phase. Similar profiles were also made in other cycle studies. The results indicated that DO was not the main reason for the two reactors showing different PAO abundances.

**Figure 4:**
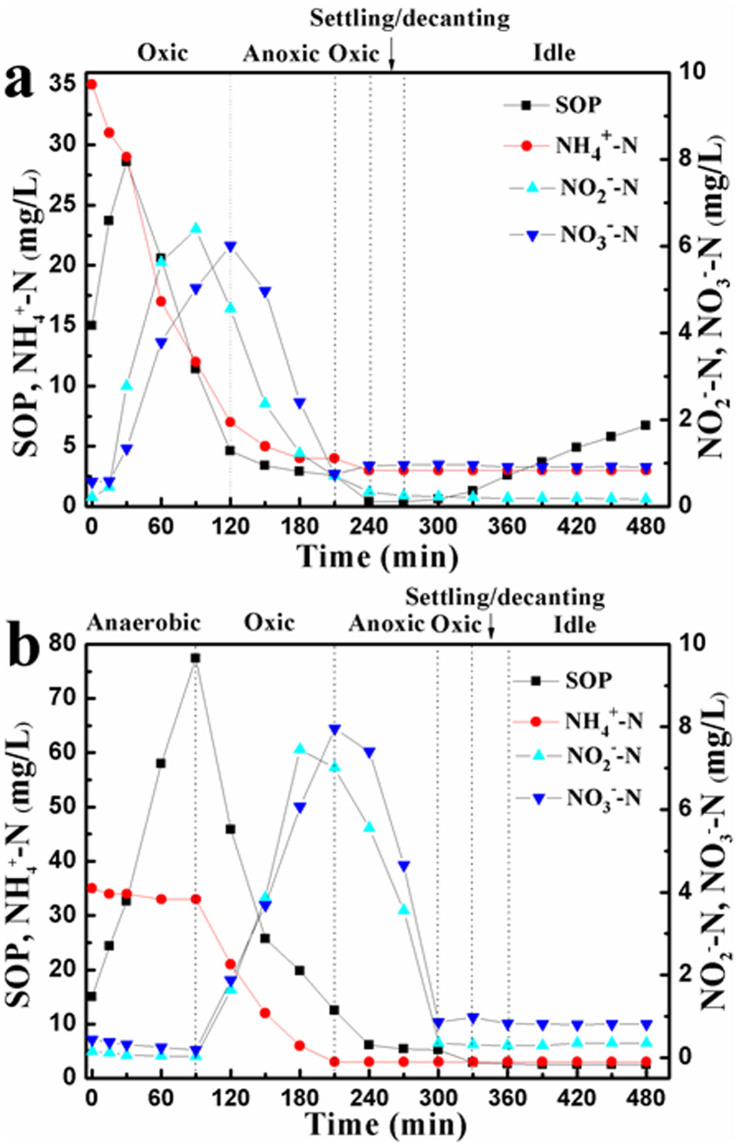
Changes of SOP, NH_4_^+^-N, NO_2_^-^-N, and NO_3_^-^-N in one typical cycle of O/A/O/EI (a) and A/O/A/O (b) reactors (on day 80).

The profile of pH change in the two reactors, however, exhibited obvious differences. In the O/A/O/EI reactor, pH gradually increased from 8.0 to 8.6 during the initial 60 min of first oxic phase and then decreased slightly during the remaining of this phase. In the following anoxic and oxic phases, a gradual increase to a final pH of 8.6 was observed. During the subsequent idle phase pH decreased gradually to the final pH of 8.2. In the A/O/A/O reactor, pH decreased from 8.0 to 7.4 in the anaerobic phase, and then a gradual increase followed by a slight decrease of pH was measured in the first oxic phase. In the subsequent anoxic phase, pH showed a gradual increase tendency then pH decreased slightly in the following oxic and idle phases. It can be clearly seen that cyclic variation of pH value in the O/A/O/EI reactor (8.0–8.6) was higher than that in the A/O/A/O reactor (7.2–8.2). The initial higher pH value achieved in the O/A/O/EI reactor might be mainly due to CO_2_ expelled from the reactor by air-stripping, while the following pH decline was probably ascribed to nitrification. It was reported that denitrification and phosphorus uptake were the primarily reasons for pH increase in the anoxic phase and oxic phase^20^. During the extended-idle phase, a slight pH decline might be owing to idle SOP release (Figure 4). There are three forms of phosphorus existed in the activated sludge: metal phosphorus via physical chemistry processes, intracellular polyphosphate inclusion, and bio-phosphorus for bacteria normal growth^21^. It is known that the amount of metal phosphorus in activated sludge is affected by pH, and higher pH value may cause higher chemical phosphorus removal^22^. However, batch tests showed that SOP removal via chemical precipitation was negligible (Table S1, supporting information), which implied SOP removal in this study was primarily due to biological effect. Previous publications showed that a high level of pH could provide a selective advantage to PAOs over other populations such as GAOs^19^,^23^. Accordingly, the higher level of cyclic pH was one reason for the O/A/O/EI reactor enriching more PAOs.

More importantly, the concentration of severe inhibitor to PAOs, i.e., FNA, is reported to closely relevant to pH value^24^. The different cyclic pH variations between the two reactors might cause different levels of FNA generation, thus the amount of FNA production between the two reactors was compared secondly. Besides pH, it is known that FNA concentration is also relevant to temperature and nitrite concentration. Temperature between the two reactors was the same (20 ± 0.5°C), and the change of nitrite as well as ammonia, nitrate, and SOP in the two reactors is shown in Figure 4. In the first oxic phase of O/A/O/EI reactor, SOP release was observed during the initial 30 min before SOP was swiftly taken up probably due to the low level of DO concentration (Fig. 3a). NH_4_^+^-N concentration was quickly decreased while NO_2_^-^-N and NO_3_^-^-N were substantially accumulated, with NO_2_^-^-N and NO_3_^-^-N accumulation up to 6.4 and 6.1 mg/L, respectively. In the subsequent anoxic phase, SOP and NH_4_^+^-N concentrations decreased slightly whereas NO_2_^-^-N and NO_3_^-^-N were largely decreased, suggesting that denitrification occurred in this period. Then, after 30 min of oxic phase (i.e., the second oxic phase), SOP, NH_4_^+^-N, NO_2_^-^-N, and NO_3_^-^-N concentrations in the effluent were 0.40, 3.0, 0.23, 0.97 mg/L, respectively. As comparison, it can be observed that a substantial amount of SOP was released in the anaerobic phase of conventional A/O/A/O reactor, then SOP uptake, NH_4_^+^-N oxidation, and NO_x_^-^-N accumulation took place concurrently. During the subsequent anoxic phase, nitrate and nitrite reductions were clearly measured. After 30 min of oxic phase (i.e., the second oxic phase), SOP, NH_4_^+^-N, NO_2_^-^-N, and NO_3_^-^-N concentration in the effluent were 2.7, 3.1, 0.29, 1.12 mg/L, respectively. Those behaviors were similar to the observations in the previous publications^25^,^26^.

It should be highlighted that the maximal nitrite accumulation in the O/A/O/EI reactor was lower than that in the conventional A/O/A/O reactor (6.4 versus 7.5 mg/L), though the two reactors had approximately same effluent nitrite concentration. In addition, it can be found that pH value at the time for the O/A/O/EI reactor achieving its maximal nitrite accumulation was higher than that for the A/O/A/O reactor (8.4–8.5 versus 7.9). According to the formula proposed by Anthonisen et al.^24^, the maximal FNA concentration generated in the O/A/O/EI reactor was about 0.52 × 10^−4^ mg HNO_2_-N/L whereas the corresponding datum was 0.24 × 10^−3^ mg HNO_2_-N/L in the A/O/A/O reactor. The maximum FNA concentration in the A/O/A/O reactor was approximately 4.6-time higher than that in the O/A/O/EI reactor. Similar observations were also observed in other cycles. It was reported that aerobic SOP uptake was severely affected when FNA was 0.26 × 10^−3^ mg HNO_2_-N/L^7^. Although the time with maximal FNA concentration was low and cyclic FNA level in the two reactors changed with time, it could be found that the average FNA level in the O/A/O/EI reactor was lower than that in the A/O/A/O reactor. In addition, batch test showed that the O/A/O/EI reactor could alleviate the inhibition of FNA on the metabolisms of PAOs even under the same FNA level, as compared with the A/O/A/O reactor (Table 2). Therefore, it can be understood that the O/A/O/EI reactor enriched more PAOs than the conventional A/O/A/O reactor. Some scientists reported that PAOs could be acclimated high nitrite and FNA concentrations when using nitrite as sole electron acceptor^27^. However, O_2_ prior to nitrite was the main electron acceptor in this study, which might be the reason for the inconsistent results.

**Figure 5:**
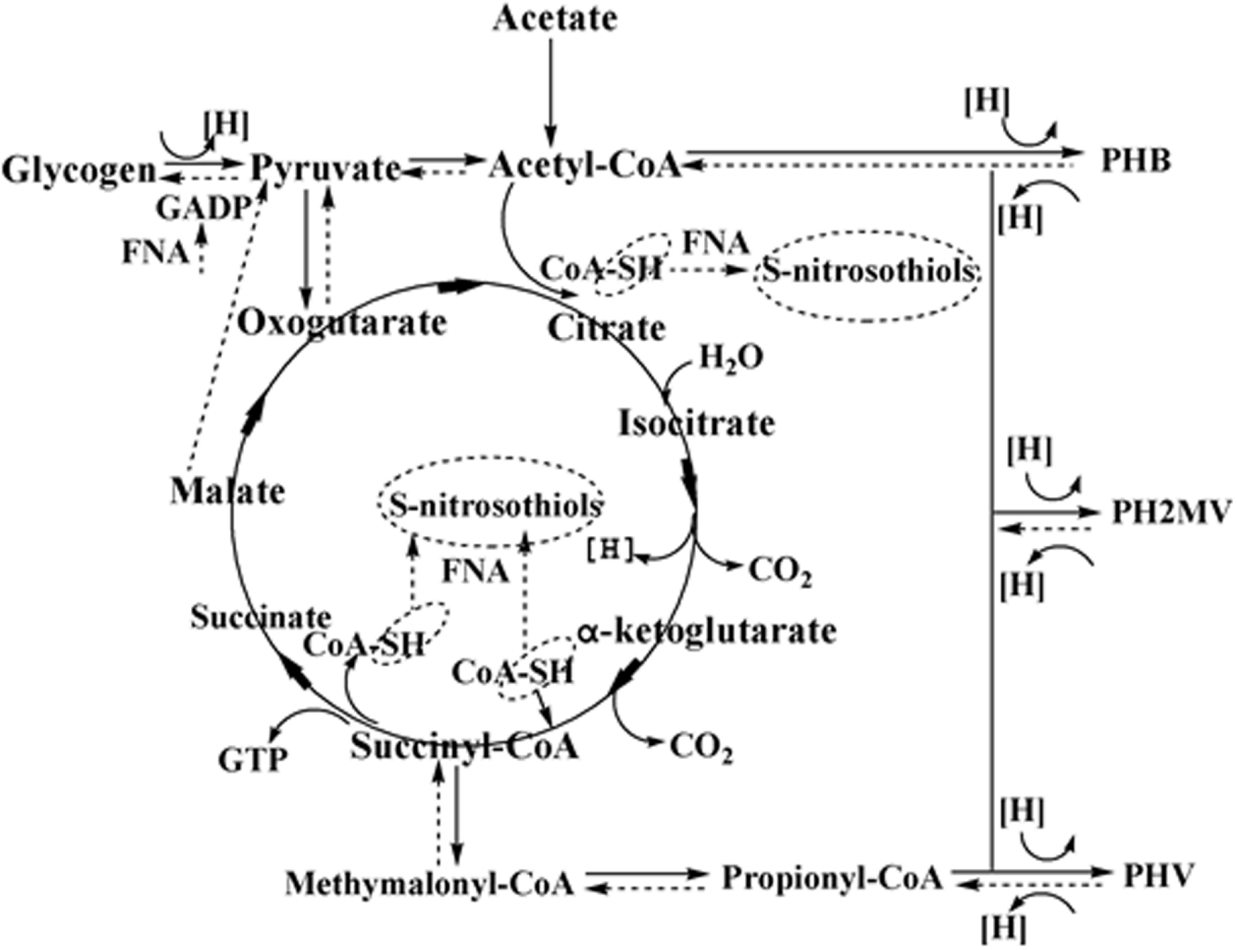
FNA inhibitory mechanisms on PAOs (dark solid line: anaerobic inhibitory mechanisms adapted from the literature^6^; dark dash line: oxic inhibitory mechanisms adapted from the literatures^4^,^5^).

FNA can inhibit or inactivate the activities of some key enzymes relevant to phosphorus removal. For instance, glyceraldehyde-3-phosphate dehydrogenase (GADP) and sulfhydryl (SH)-containing enzymes, which are respectively key enzymes involved in both glycolysis (gluconeogenesis) and the tricarboxylic acid (TCA) cycle, are reported to be heavily inhibited through reaction with FNA (Figure 5). The transformations of key metabolic intermediates such as glycogen and PHAs are closely related to glycolysis (gluconeogenesis) and the TCA cycle, thus the activity or abundance of PAOs will be reduced when FNA interferes with the pathways of glycolysis (gluconeogenesis) or the TCA cycle. From the “Methods” section, it can be found some differences between the two reactors. In the conventional A/O/A/O reactor, acetate is consumed in the anaerobic phase whereas it is taken up aerobically in the new O/A/O/EI reactor. This different metabolic behavior will cause certain metabolic differences, which might be one reason for the O/A/O/EI reactor enriching higher PAOs. Also, this different metabolic behavior might result in different metabolic responses of PAOs to FNA. For example, compared with the conventional A/O/A/O reactor where ATP and NADH_2_ for PHAs formation were respectively provided via poly-P hydrolysis and glycogen degradation, the TCA cycle seems to supply both ATP and NADH_2_ for PHAs synthesis in the O/A/O/EI reactor since it is generally accepted that the TCA cycle will dominate under aerobic conditions. However, it is still unclear why the O/A/O/EI reactor can alleviate the inhibition of FNA on the metabolisms of PAOs, as the TCA cycle plays an important role in PAO metabolisms of both regimes. Further efforts need to be carried out in future.

### Comparison with other strategies for minimizing the inhibitory effect of FNA on PAOs

This paper presents an effective strategy for mitigating the generation of FNA and its inhibition on PAOs. That is, by modifying the wastewater treatment operation regime as the O/A/O/EI regime the abundance of PAOs and the efficiency of phosphorus removal can be significantly improved. This was experimentally demonstrated via a long-term test in two reactors operated as the new O/A/O/EI regime and the conventional A/O/A/O regime, respectively. The abundance of PAOs cultured in the O/A/O/EI reactor was about 11% higher than those in the conventional A/O/A/O reactor, which led to 15% of improved phosphorus removal efficiency. Moreover, this wastewater treatment regime based strategy did not decrease but slightly increase the nitrogen removal performance. Considering the huge quantities of wastewater treated daily, this strategy has a significant consequence from an ecological perspective.

Compared with other strategies such as pH adjustment and temperature control^6^,^15^, this wastewater treatment regime based strategy does not require consumption of any additional chemicals and energy, which makes this strategy more economical and practical. This strategy can also integrate with the step-feeding mode easily, a practically effective method for minimizing the inhibitory effects of FNA, to gain a better nutrient removal performance. It was reported that step-feeding modes could greatly reduce the FNA inhibition influence as compared to dump-feeding^28^. By modifying the influent mode of O/A/O/EI regime, this wastewater treatment regime based strategy can easily combine with the feeding based strategy, which may cause further reduction of FNA inhibitory. Therefore, the strategy presented here might provide a practically promising solution to the “nitrogen-phosphorus challenge” faced by WWTPs. Furthermore, the enrichment of PAOs in the O/A/O/EI reactor is driven by the O/EI regime. It was reported that the O/EI regime could achieve very good phosphorus removal readily and steady when using glucose, a substrate usually considered being detrimental for PAO proliferations, as the sole carbon source^30^. Thus, the O/A/O/EI process reported in this paper may provide an ideal technology for BNR removal from carbohydrate-rich wastewaters. Generally, glucose or other carbohydrate compounds in domestic wastewater are at low levels, because it can be readily bio-fermented to volatile fatty acids in sewer systems. However, in some WWTPs where industrial or agricultural factories discharging carbohydrate-rich wastewaters are located nearby, or in some specific areas where the distance between the wastewater discharge sources and wastewater treatment unit is short (e.g., the highway rest areas, one of our parallel researches), wastewater carbohydrate may maintain at high levels. In these areas, the O/A/O/EI process may have an excellent application perspective, and the batch-scale study presented here may provide a useful reference for designs in future.

It should be noted that although the hydraulic retention time (HRT) between the O/A/O/EI and A/O/A/O reactors operated in this studies was maintained the same, the HRT controlled in the O/A/O/EI regime may slightly higher than that in the conventional BNR regime, because one cycle of the conventional BNR systems can be shortened to be 6 h via process optimization whereas the O/A/O/EI regime needs a relatively long idle period to enrich PAOs (e.g., 210 min). This characteristic implies that the proposed O/A/O/EI regime will increase the volumes of bioreactors when treating the same amount of wastewater. However, this drawback can be settled via reactor reconfiguration as proposed in Figure S1. Despite that this new strategy was demonstrated using sequencing batch reactors in this study (due to the availability of the equipment), it has also the potential to be applied in a continuous system. For a continuous-flow activated sludge system, an extra reactor for regurgitant sludge rest (3.5 h of the retention time seems to be enough) is required to set up in the side-stream for the enrichment of PAOs, and the construction invest of extra side-stream reactor is low, as compared with other strategies. It should also be emphasized that full-scale tests are required to fully evaluate the feasibility and potential of this strategy though excellent results have already obtained in our laboratory experiments.

## Methods

### Synthetic wastewater

Synthetic wastewater used throughout these investigations, unless otherwise described, was the same and prepared daily. Acetate was used for the sole carbon source since it was the most common volatile fatty acids present in real domestic wastewaters^29^. KH_2_PO_4_ was selected as the phosphorus source. The chemical oxygen demand (COD) and orthophosphate (PO_4_^3-^-P) concentrations in the wastewater were approximately maintained at 300 and 15 mg/L, respectively. Hence, the ratio of COD: PO_4_^3-^-P in the influent was controlled at 20 mg COD/(mg PO_4_^3-^-P), which was considered as being favorable to the growth of PAOs^2^. The concentrations of the other nutrients in the synthetic wastewater were the same and indicated below (per liter): 133.8 mg NH_4_Cl, 0.5 mg CaCl_2_, 0.5 mg MgSO_4_, and 1 mL of a trace metals solution. The trace metals solution had been described in our previous publication^30^.

### Operation of the new and conventional four-step BNR processes

This study was conducted in two identical sequencing batch reactors with a working volume of 12 L each. Both reactors were seeded with activated sludge obtained from a WWTP in Changsha, PR China, which was operated as A^2^/O process. The initial concentration of mixed liquor suspended solids (MLSS) was 3800 mg/L and mixed liquor volatile suspended solid (MLVSS) was 2400 mg/L. The activated sludge was maintained at 20 ± 0.5°C in a temperature controlled room. One reactor was performed as the developed O/A/O/EI regime while the other was operated as the classical four-step A/O/A/O regime in parallel. Both reactors were operated with three 8-h cycles daily, and each 8-h cycle of the O/A/O/EI regime consisted of a 120 min oxic phase, a 90 min anoxic phase, a 30 min oxic phase, a 30 min settling and decanting phase, and a 210 min idle phase. As comparison, the conventional four-step BNR regime was also operated according to the literature with minor revision^31^,^32^, and each cycle of this regime contained a 90 min anaerobic period, a 120 min aerobic period, a 90 min anoxic period, a 30 min aerobic period, a 30 min settling/decanting period, and a 120 min idle period. For each cycle, certain volume supernatant was discharged from both reactors after the settling phase and was replaced with synthetic wastewater during the initial 5 min of first oxic phase (the O/A/O/EI reactor) and anaerobic phase (the A/O/A/O reactor), respectively. The HRT and sludge retention time (SRT) in the two reactors were controlled at approximately 16 h and 20 d, respectively. During anaerobic phase, the A/O/A/O reactor was mixed with a mechanical stirrer (150 rpm) while during the aerobic phase, air was supplied into both reactors at a flow rate of 15 L/min. The initial pH level in both reactors was controlled at 8.0 by adding 0.5 M HCl or 0.5 M NaOH solutions.

It should be noted that during the idle phase mixture stirring was not conducted in the routine operation of both reactors, but when cyclic tests were carried out, both reactors were mixed with a mechanical stirrer (150 rpm) to facilitate sampling. The mixed liquor samples were taken every 30 min and immediately filtered through a Whatmann GF/C glass microfiber filter (1.2 μm). The sludge sample was used to assay for MLSS, MLVSS, PHAs, and glycogen. The filtrate was used for the analyses of SOP, COD, NH_4_^+^-N, and NO_x_^-^-N.

### Phosphorus removal test via chemical precipitation at different pH

Phosphorus can be removed via chemical precipitation when some metal ions such as Ca^2+^, Mg^2+^ are present in wastewater. With the increase of pH the chemical phosphorus precipitation was enhanced^22^. Hence, one batch test was performed without the activated sludge microorganisms to assess the effect of pH on chemical phosphorus precipitation. Firstly, 15 L synthetic wastewater mentioned above was divided evenly into 5 identical reactors with working volumes of 3.0 L each. Then, the reactors were added 0.5 M HCl or 0.5 M NaOH solutions to keep the pH value 6.5, 7.0, 7.5, 8.0, and 8.5, respectively, the other operational conditions were the same as the O/A/O/EI reactor described above expect that there was no sludge microorganisms. Finally, SOP in the supernatant of the 5 reactors was detected after several cycles. Therefore, it is readily to assess the effect of pH on chemical phosphorus precipitation via measuring the SOP concentration in supernatant.

### Comparison of the effect of different FNA levels on PAO metabolisms between the two reactors

The A/OA/O reactor is developed from the conventional A/O phosphorus removal regime whereas the O/A/O/EI reactor is developed from the recently exploited O/EI regime, thus it is necessary to investigate whether there are different effects on PAO metabolisms between the two reactors even under the same FNA levels. The following batch experiment was executed to provide such support. Two identical sludge mixtures (2.4 L each) were respectively withdrawn from a WWTP in Changsha, PR China. The mixtures were centrifuged (5000 rpm for 5 min) and washed three times with tap water to remove the residual NH_4_^+^-N, NO_x_^-^-N, SOP, and COD. Then, they were resuspended in tap water with a final volume of 1.2 L each before being evenly divided into six reactors. The two groups of reactors (six each) were respectively operated as the same as the A/OA/O and O/A/O/EI reactors expect for the following differences. Allyl-Nthiourea (a nitrification inhibitor) was added at a concentration of 2 mg/L to each reactor to inhibit the nitrification according to the literature^33^. Temperature was controlled at 20 ± 0.5°C and pH was on-line controlled consistently at pre-designed set-point (pH = 8.0 ± 0.1) by a programmable logic controller using 0.5 M HCl solution and 0.5 M NaOH solution. Thus, the FNA concentrations of six reactors for each group were respectively controlled at 0, 0.05 × 10^−3^, 0.15 × 10^−3^, 0.26 × 10^−3^, 0.38 × 10^−3^, and 0.51 × 10^−3^ mg HNO_2_-N/L through controlling nitrite concentration, pH, and temperature. It was reported that the FNA concentration could be calculated by the formula S_N-NO2_/(Ka × 10^pH^) with K_a_ value determined by the formula K_a_ = e^(−2300/(T + 273))^ for a given temperature T (°C)^24^. When effluent SOP concentration among these reactors reached stable, cyclic studies were performed and the data were reported.

### Chemical and microbial analyses

COD, SOP, nitrite, nitrate, ammonia, MLSS, and MLVSS were measured by standard methods^34^. The determinations of glycogen, poly-3-hydroxybutyrate (PHB), poly-3-hydroxyvalerate (PHV), and poly -3 -hydroxy -2- methylvalerate (PH2MV) were measured according to our previous publication^30^. The PHAs were the summation of PHB, PHV, and PH2MV.

The fluorescence in situ hybridization (FISH) with 16s rRNA-targeted oligonucleotide probes was carried out to quantify the abundances of PAOs and GAOs, and the methods were the same as described in the literature^17^. Briefly, sludge samples were taken and fixed in 4% formaldehyde for 20 h at 4°C and then subjected to freeze-thaw treatment in order to enhance the penetration of oligonucleotide probes. Cell samples were attached to poly-L-lysine coated slides and dehydrated with ethanol. The following hybridization and washing procedures were the same as that in the literature^35^. For quantitative analysis, 20 microscopic fields were analyzed for the hybridization of individual probes using a confocal scanning laser microscope (FV 500) with image database software (VideoTesT Album3.0). The oligonucleotide probes specific for PAOs, GAOs, and total bacteria, which were respectively labeled with 5′AMCA, 5′Cy3, and 5′FITC, were listed in Table S2.

## Author Contributions

J.W.Z. carried out the experiments and drafted the paper, D.B.W. and X.M.L. designed the experimental plan and revised the paper, Q.Y., H.B.C., Y.Z., H.X.A. and G.M.Z. analyzed the data. All authors contributed to the scientific discussion.

## Supplementary Material

Supplementary Information Supporting Information

## Figures and Tables

**Table 1:** Summary of reactor performances of the O/A/O/EI and A/O/A/O reactors during steady-state operation^a^

Item	O/A/O/EI reactor	A/O/A/O reactor
Effluent SOP (mg/L)	0.41 ± 0.11	2.70 ± 0.18
SOP removal efficiency (%)	97 ± 0.73	82 ± 1.2
Effluent NH_4_^+^-N (mg/L)	2.68 ± 0.41	3.75 ± 0.15
Effluent NO_2_^-^-N (mg/L)	0.23 ± 0.08	0.31 ± 0.03
Effluent NO_3_^-^-N (mg/L)	0.85 ± 0.21	1.21 ± 0.09
Effluent pH	</city>8.6 ± 0.2	8.1 ± 0.3
TN removal efficiency (%)	89 ± 1.8	85 ± 2.1

^a^Results are the average and standard deviation, and the data were obtained during the steady-state operation.

**Table 2:** Effect of FNA on the phosphorus removal performance of O/A/O/EI and A/O/A/O reactors^a^

Influent FNA (×10^−3^ mg HNO_2_-N/L)	O/A/O/EI reactor				A/O/A/O reactor				
	PHA-up/VFA (C-mol/C-mol)	Gly-syn/VFA (mM-C/g VSS)	Effluent SOP (mg/L)	Idle SOP release (mg/L)	Anaerobic SOP release (mg/L)	Gly-de/VFA (C-mol/C-mol)	PHA-up/VFA (C-mol/C-mol)	Gly-syn (C-mol/g VSS)	Effluent SOP (mg/L)
0	0.52 ± 0.03	0.51 ± 0.04	0.22 ± 0.03	6.89 ± 0.04	62 ± 0.7	0.62 ± 0.04	1.19 ± 0.02	1.87 ± 0.09	0.75 ± 0.05
0.05	0.48 ± 0.05	0.49 ± 0.05	0.39 ± 0.05	6.22 ± 0.06	58 ± 1.2	0.61 ± 0.03	1.06 ± 0.05	1.72 ± 0.05	1.04 ± 0.07
0.15	0.42 ± 0.02	0.47 ± 0.08	1.15 ± 0.07	5.79 ± 0.03	53 ± 0.9	0.59 ± 0.05	1.01 ± 0.04	1.59 ± 0.07	3.85 ± 0.09
0.26	0.38 ± 0.05	0.41 ± 0.04	1.55 ± 0.11	5.42 ± 0.06	49 ± 0.8	0.55 ± 0.02	0.92 ± 0.06	1.36 ± 0.04	4.05 ± 0.11
0.38	0.35 ± 0.06	0.40 ± 0.02	2.36 ± 0.05	5.18 ± 0.02	43 ± 1.4	0.54 ± 0.03	0.79 ± 0.07	1.24 ± 0.06	5.12 ± 0.12
0.51	0.31 ± 0.03	0.38 ± 0.03	5.10 ± 0.08	4.87 ± 0.05	35 ± 0.5	0.53 ± 0.04	0.71 ± 0.05	1.12 ± 0.07	7.05 ± 0.09

^a^Results are the average and standard deviation, and the data were obtained during the steady-state operation.
