# Supplementary material for: An efficient process for wastewater treatment to mitigate free nitrous acid generation and its inhibition on biological phosphorus removal
Source: Sci Rep. 2015 Feb 27;5:8602. doi: 10.1038/srep08602 (PMC4342570; doi:10.1038/srep08602)
Supplement: Supplementary Information — Supporting Information [file srep08602-s1.doc]

**Supporting Information**

**An efficient process for wastewater treatment to mitigate free nitrous acid generation and its inhibition on biological phosphorus removal**

Jianwei Zhao ^1,2^, Dongbo Wang 1,2,3,4,5[[1]](#footnote-2)*, Xiaoming Li ^1,2*^, Qi Yang ^1,2^, Hongbo Chen ^1,2^, Yu Zhong ^1, 2^, Hongxue An ^1, 2^, Guangming Zeng^1,2^

^1^ College of Environmental Science and Engineering, Hunan University, Changsha 410082, China

^2^ Key Laboratory of Environmental Biology and Pollution Control, Hunan University, Ministry of Education, Changsha 410082, China

^3^ State Key Laboratory of Pollution Control and Resources Reuse, School of Environmental Science and Engineering, Tongji University, 1239 Siping Road, Shanghai 200092, China

^4^ Advanced Water Management Centre, The University of Queensland, QLD 4072, Australia

^5^ Jiangsu Tongyan Environmental Production Science and Technology Co. Ltd., Yancheng 224000, China

Table S1. Effect of pH on phosphorus removal via chemical precipitation in this study.

|  | pH6.5 | pH7.0 | pH7.5 | pH8.0 | pH8.5 |
| --- | --- | --- | --- | --- | --- |
| Influent SOP (mg/L) | 14.8±0.3 | 15.1±0.1 | 15.0±0.2 | 14.8±0.2 | 15.0±0.3 |
| Residual SOP in the supernatant (mg/L) | 14.7±0.1 | 15.0±0.1 | 14.8±0.3 | 14.6±0.1 | 14.7±0.1 |
| Average SOP removed by precipitation (%) | 0.6 | 0.6 | 1 | 1.3 | 2 |

^a^ Results are the averages and their standard deviations of triplicate tests.

| Table S2. Oligonucleotide Probes Specific for PAO, GAO, and Total Bacteria Used in This Study | | | |
| --- | --- | --- | --- |
| Probe | Sequence5’-3’ | Specifity | Reference |
| EUB338-І | GCT GCC TCC CGT AGG AGT | Most bacteria | 1 |
| EUB338-II | GCA GCC ACC CGT AGG TGT | Planctomycetales | 2 |
| EUB338-III | GCT GCC ACC CGT AGG TGT | Verrucomicrobiales | 2 |
| PAO462 | CCG TCA TCT ACW CAG GGT ATT AAC | Most Accumulibacter | 3 |
| PAO651 | CCC TCT GCC AAA CTC CAG | Most Accumulibacter | 3 |
| PAO846 | GTT AGC TAC GGC ACT AAA AGG | Most Accumulibacter | 3 |
| GAOQ431 | TCC CCG CCT AAA GGG CTT | Some Competibacter | 4 |
| GAOQ989 | TTC CCC GGA TGT CAA GGC | Some Competibacter | 4 |
| GB_G2 | TTC CCC AGA TGT CAA GGC | Some Competibacter | 5 |
| TFO-DF218 | GAA GCC TTT GCC CCT CAG | ‘Defluviicoccus’-related organisms | 6 |
| TFO-DF618 | GCC TCA CTT GTC TAA CCG | ‘Defluviicoccus’-related organisms | 6. |
| DF988 | GAT ACG ACG CCC ATG TCA AGG G | ‘Defluviicoccus’-related organisms | 7 |
| DF1020 | CCG GCC GAA CCG ACT CCC | ‘Defluviicoccus’-related organisms | 7 |


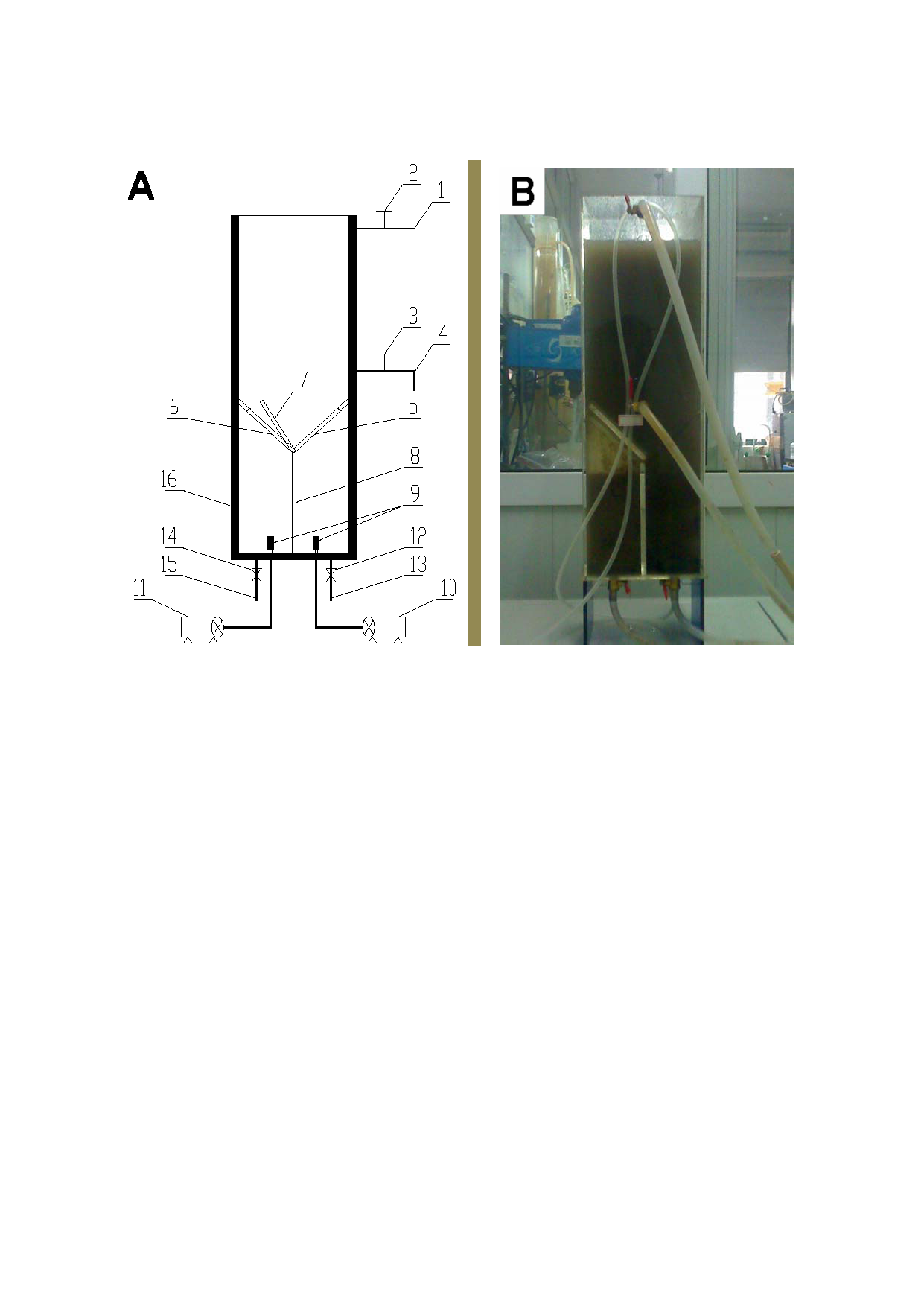


b

1-intake pipe; 2-inlet valve; 3-emptying valve; 4-drainage pipe; 5-supporting incline I; 6-supporting incline II; 7-rolling plank; 8-vertical plank; 9-air diffuser; 10-air compressor I; 11-air compressor II; 12-sludge discharging valve I; 13-sludge discharge pipe I; 14-sludge discharging valve II; 15- sludge discharge pipe II; 16-STH-SBR body

a

Figure S1. The schematic diagram (a) and photograph (b) of the proposed reactor reconfiguration. The main difference between the proposed sequencing batch reactor and conventional sequencing batch reactor was the configuration of sludge tank. The sludge tank of between the proposed sequencing batch reactor was divided equally into two tanks (each contained a sole air diffuser) by a vertical plank, and a rolling plank was connecting with the top of vertical plank. By rotating the rolling plank, activated sludge in the two tanks can easily alternate to mix with wastewater. This reactor can operated 4 cycles daily (6h per cycle) which thereby save reactor volume largely^8^.

**References**

1. Amann, R., Ludwig, W. & Schleifer, K.H. Phylogenetic identification and in situ detection of individual microbial cells without cultivation. *Microbiol. Rev.* **59**, 143-169 (1995).

2. Daims, H. *et al*. The domain-specific probe EUB338 is insufficient for the detection of all bacteria: development and evaluation of a more comprehensive probe set. Syst. *Appl. Microbiol*. **22**, 434-444 (1999).

3. Crocetti, G.R. *et al*. Identification of polyphosphate-accumulating organisms and design of 16S rRNA-directed probes for their detection and quantitation. *Appl. Environ. Microbiol*. **66**, 1175-1182 (2000).

4. Crocetti, G.R., Banfield, J.F., Keller, J., Bond, P.L. & Blackall, L.L. Glycogen-accumulating organisms in laboratory-scale and full-scale wastewater treatment processes. *Microbiology* **148**, 3353-3364 (2002).

5. Kong, Y. H., Ong, L., Ng, W. J. & Liu, W. T. Diversity and distribution of a deeply branched novel proteobacterial group found in anaerobic-aerobic activated sludge processes. *Environ. Microbiol.* **4,** 753-757 (2002).

6. Wong, M.T., Tan, F.M., Ng, W.J. & Liu, W.T., 2004. Identification and occurrence of tetrad-forming Alphaproteobacteria in anaerobic–aerobic activated sludge processes. *Microbiology* **150**, 3741-3748 (2004).

7. Meyer, R.L., Saunders, A.M. & Blackall, L. L. Putative glycogen-accumulating organisms belonging to the Alphaproteobacteria identified through rRNA-based stable isotope probing. *Microbiology* **152**, 419-429 (2006).

8. Wang, D. *et al*. A new configuration of sequencing batch reactor operated as a modified aerobic/extended-idle regime for simultaneously saving reactor volume and enhancing biological phosphorus removal. *Biochem. Eng. J.* **87**, 15-24 (2014).

1. ^*^ Corresponding author. Tel.: +86-731-88823967; fax: +86-731-88822829.

   E-mail addresses: w.dongbo@yahoo.com (D. Wang), xmli@hnu.edu.cn (X. Li). [↑](#footnote-ref-2)
